# Development of a Competitive Cystatin C-Specific Bioassay Suitable for Repetitive Measurements

**DOI:** 10.1371/journal.pone.0147177

**Published:** 2016-01-22

**Authors:** Tatjana Damm, Holger Spiegel, Stefan Barth, Rainer Fischer, Joerg Naehring

**Affiliations:** 1 Fraunhofer Institute for Molecular Biology and Applied Ecology IME, Aachen, Germany; 2 Institute for Molecular Biology, RWTH Aachen University, Aachen, Germany; 3 Institute of Applied Medical Engineering, RWTH Aachen University, Aachen, Germany; Central South University, CHINA

## Abstract

Human cystatin C (hCC), a cysteine protease inhibitor, has been proposed as a diagnostic marker because its serum levels correlate with certain cardiovascular and kidney diseases. All current hCC assays are based on *ex vivo* detection. Here we describe the generation and evaluation of antibodies that allow the repetitive binding and release of hCC and hCC-fusion proteins, a prerequisite for long-term measurement, which is required for compatibility with implantable biochip devices and for the development of innovative antibody-based assays suitable for continuous *in vivo* and *in vitro* monitoring. Recombinant hCC and hCC-fusion proteins were produced in *Escherichia coli* and HEK293T cells and were used to generate antibodies by hybridoma technology. After screening by indirect and sandwich ELISAs, 12 monoclonal hybridoma cell lines producing hCC-specific monoclonal antibodies were identified. To determine their hCC association and dissociation properties, the antibodies were analysed by surface plasmon resonance spectroscopy, revealing three with the desired fast binding and moderate-to-fast release characteristics. The analysis of binding and dissociation in the presence of hCC and hCC-fusion proteins using fluorescence-based replacement assays showed that mAb CyDI-4 was the most suitable for further analysis. The results showed that repetitive replacement on mAb CyDI-4 was possible and that most of the change in signal intensity occurred after 20–30 min. Furthermore, the suitability of mAb CyDI-4 for serum hCC measurement was confirmed by a fluorescence-based replacement assay using serially-diluted reference serum from the Institute for Reference Materials and Measurements (ERM-DA471/IFCC). Our results suggest that the assay covers the physiological and pathological ranges of hCC.

## Introduction

Human cystatin C (hCC) is a basic 13-kDa protein from the cysteine protease inhibitor family which was discovered in 1961 [1]. The protein is produced by most nucleated cells [2], and is removed from the blood by glomerular filtration and reabsorbed by the proximal tubules [3] where it is degraded [4]. By 1985, the serum concentration of hCC was proposed as a marker for the estimated glomerular filtration rate (eGFR), which is an indicator of kidney health [5]. Although creatinine is used more frequently for clinical diagnosis, hCC was considered a more accurate eGFR marker because its abundance was thought to be independent of height, gender, age and muscle mass [6]. However, it is now known that hCC is not a completely independent diagnostic marker.

Several equations have been developed to overcome limitations caused by the dependencies of hCC and creatinine [7]. There is still no consensus as to whether the eGFR is best predicted by equations based on hCC (eGFRcys) or creatinine (eGFPcr), and the accuracy seems to depend on the type of disease [8–13]. Recently a combined equation (eGFPcys-cr) was shown to provide superior predictions than either individual marker alone in chronic kidney disease [14]. There are also data that suggest hCC participates in protective mechanisms against neurodegenerative diseases [15,16]. In Alzheimer’s disease, hCC has been shown to inhibit the aggregation of amyloid beta but not to dissolve pre-formed aggregates [17]. In cardiovascular disease, elevated hCC concentrations in serum are associated with higher risk factors [18,19].

The first hCC detection method was an enzyme-linked immunosorbent assay (ELISA) based on polyclonal rabbit antibodies [20]. Diverse ELISA kits are now available to measure hCC concentrations in body fluids, as well as the automated particle enhanced turbidimetric immunoassay (PETIA) [21] and the particle enhanced nephelometric immunoassay (PENIA). All current hCC assays for serum and other body fluids are based on *ex vivo* detection. However, *in vivo* diagnostics and therapies are emerging. Patients treated with implantable devices for cardiac rhythm management could be fitted with an additional diagnostic tool that monitors their health by the continuous *in vivo* measurement of hCC concentrations in the blood stream. In addition to suitable hardware solutions this would require the development of appropriate reusable detection reagents for *in vivo* applications.

Here we report the generation and characterisation of hCC-specific antibodies that allow the development of *in vivo* monitoring assays based on the repetitive binding and release of hCC and hCC-fusion proteins. The suitability of the antibodies was investigated in detail by surface plasmon resonance (SPR) spectroscopy and fluorescence-based replacement assays. We discuss our findings in the context of the development of an hCC-specific bioassay compatible with diagnostic implants.

## Results

### Cloning, production and purification of hCC, hCC-fusion proteins and GST

The hCC amino acid sequence (GenBank CAA36497.1) was randomly reverse translated and the resulting sequence was codon optimised for *Escherichia coli* and *Homo sapiens* (http://gcua.schoedl.de/, http://www.kazusa.or.jp/codon/). The synthetic gene was prepared by Eurofins MWG Operon and the coding sequence is provided in the Supplementary Information. The sequences for hCC, the hCC-fusion proteins and GST were assembled by splice overlap extension (SOE)-PCR, standard PCR and *in vivo* amplification (data not shown).

**Fig 1:**
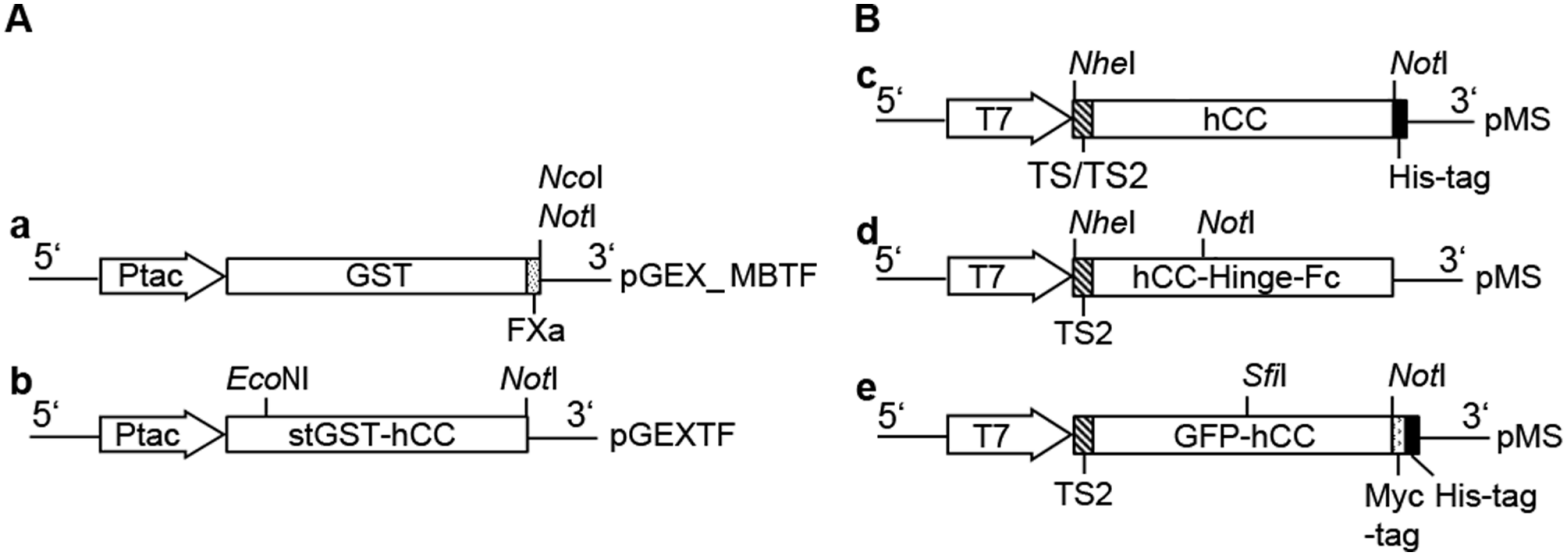
GST and stGST-hCC prokaryotic expression cassettes. Prokaryotic expression cassettes for production of GST (**a**) and stGST-hCC (**a**) in the pGEX5x3-based vector (**pGEX_MBTF**, **pGEXTF**) and eukaryotic expression cassettes for the production of hCC (**c**), hCC-Hinge-Fc (**d**), **and** GFP-hCC (**e**) in the pMS-vector (**pMS**, Stoecker *et al*. 2003). **Ptac:** tac promoter, **FXa:** Factor Xa cleavage site, **GST:** Sequence encoding glutathione-S-transferase, **stGST-hCC:** Sequence coding for the stabilised fusion of GST and human cystatin C (hCC), ***Nco*I, *Nhe*I, *Not*I, *Sfi*I:** Restriction sites, **T7:** T7 promoter/priming site, **TS:** Native secretion signal of hCC, **TS2:** Secretion signal from the V-J2-C region of the mouse Igκ, **His-tag:** Sequence encoding six histidine residues, **Myc-tag:** Sequence encoding c-Myc, **hCC:** Codon-optimised sequence encoding the human cystatin C, **hCC-Hinge-Fc:** Sequence encoding a fusion protein comprising hCC, the hinge region and fragment crystallisable of a human antibody, **hCC-SNAP:** Sequence encoding a fusion protein comprising hCC and mutant O^6^-alkylguanine-DNA alkyltransferase, **GFP-hCC:** Sequence encoding a fusion protein comprising enhanced green fluorescent protein and hCC.

The cassettes for prokaryotic and eukaryotic protein expression are shown in Fig 1. We used glutathione-S-transferase (GST), enhanced green fluorescent protein (GFP) and the immunoglobulin hinge region and crystallisable fragment (hinge-Fc) as fusion partners. GST and the stGST-hCC fusion protein were produced in *E*. *coli* (the fusion protein was named stGST-hCC because it represents a stabilised form of the protein, lacking the original intrinsic factor Xa cleavage site) and were purified by affinity chromatography using Glutathione HiCap Matrix (Quiagen, Hilden, Germany). The remaining proteins were produced in HEK293T cells and purified by Protein A affinity chromatography (Protein A Ceramic HyperD^®^ F, Pall Corporation, Dreieich, Germany) or immobilised metal ion affinity chromatography (IMACT; Ni-NTA Superflow Cartridges, Qiagen). The hCC protein was purified further by anion exchange chromatography (AEC; Q Ceramic HyperD^®^ F, Pall Corporation). The pure proteins were analysed by discontinuous sodium dodecylsulfate polyacrylamide gel electrophoresis (SDS-PAGE) and immunoblotting (data not shown). The concentration of the protein samples was determined using a bicinchoninic acid (BCA) assay kit (Pierce, Thermo Fisher Scientific, Waltham, USA). The concentration of the target protein was calculated according to the purity, as determined by image analysis using AIDA software (Raytest, Straubenhardt, Germany) on SDS-PAGE gels stained with Coomassie Brilliant Blue.

### Immunisation, hybridoma generation and screening

The hCC-specific antibodies were generated by immunising three BALB/c mice with stGST-hCC (60 μg/30 μg antigen regime with GerbuMM (GERBU Biotechnik GmbH, Heidelberg, Germany) as the adjuvant). The development of hCC-specific antibodies was monitored by measuring the titres on days 25 and 31. We also determined the titre of antibodies against hCC and GST. The titre was defined as the highest dilution step that achieved double the background absorption value. The first (day 25) titres were in the range 1: 6400–1: 25 600 and the second (day 31) titres were in the range 1: 51 200–1: 102 400. Prior to hybridoma generation, the mice were boosted three times with antigen only on three successive days. After hybridoma generation and growth, the hybridoma supernatant was analysed by ELISA for the presence of hCC-specific binders. An additional protein (hCC-Hinge-Fc) was selected for screening, allowing us to exclude non-binders and neotope binders. Limiting dilution followed by screening was used to produce monoclonal cell lines from positive hybridoma cell cultures. Ultimately, 12 monoclonal hybridoma cell lines were identified, producing antibodies specific for hCC. These antibodies were analysed by SPR spectroscopy for their hCC binding and dissociation characteristics.

### SPR-based analysis of antibodies from monoclonal hybridoma supernatants

**Fig 2:**
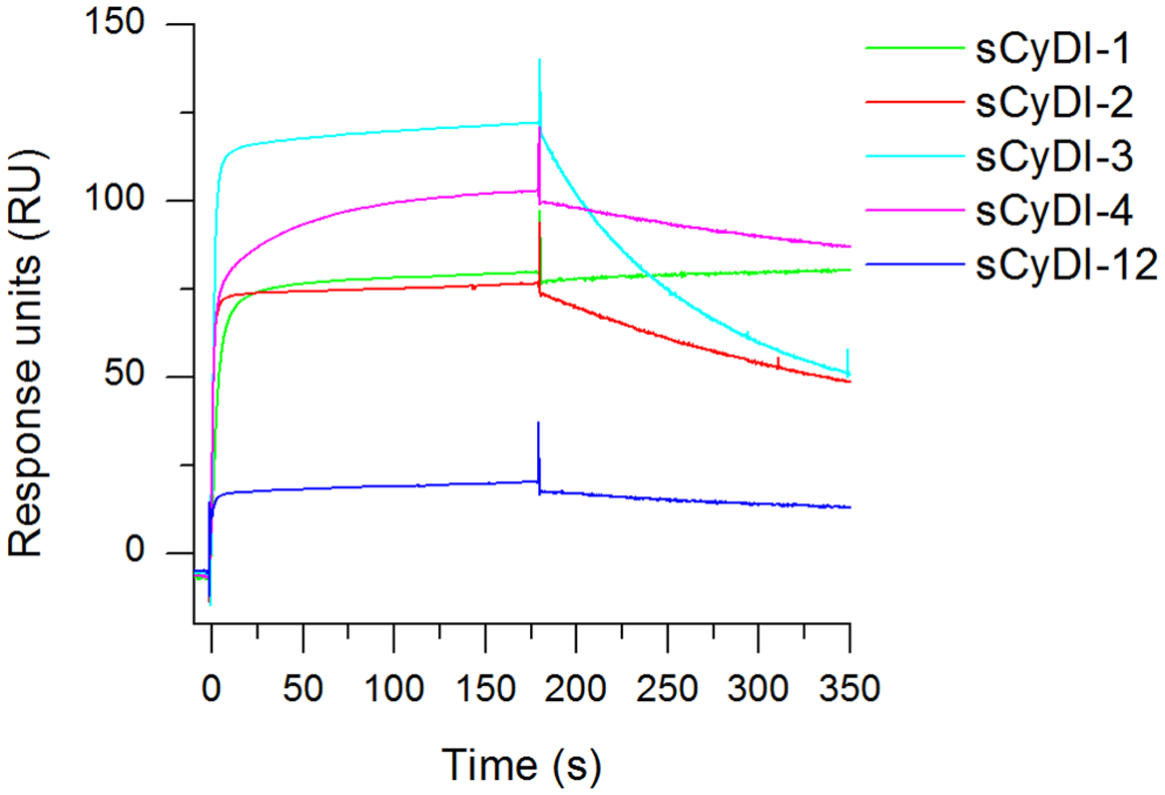
SPR analysis of antibodies from hybridoma supernatants based on their ability to bind and release hCC. Antibodies from monoclonal hybridoma supernatants were analysed according to their hCC binding (180 s) and dissociation characteristics. The supernatant was pre-diluted 1:6 in PBS. The responses resulting from the capture of hCC by five antibodies are shown in different colours. The antibodies originated from three different hybridoma generations and their capture level differed according to their abundance in the supernatant.

Hybridoma supernatants were analysed by SPR spectroscopy using the Biacore™ T200 system (GE Healthcare, Little Chalfont, UK). For kinetic analysis, antibodies were captured on a RAM-Fc chip followed by the injection of recombinant hCC (18 μg/ml). The antibody capture levels varied according to the amount of antibody in the sample. From 12 initial samples, five showed appropriate binding properties and differed in dissociation characteristics (Fig 2). The dissociation was fastest for monoclonal antibody (mAb) CyDI-3, followed by mAbs CyDI-2, CyDI-4 and CyDI-12. No dissociation was detected for mAb CyDI-1 during the assay. Precise kinetic constants were not determined because the purpose of the experiment was the direct comparison of the available antibodies.

### Antibody production and purification

**Fig 3:**
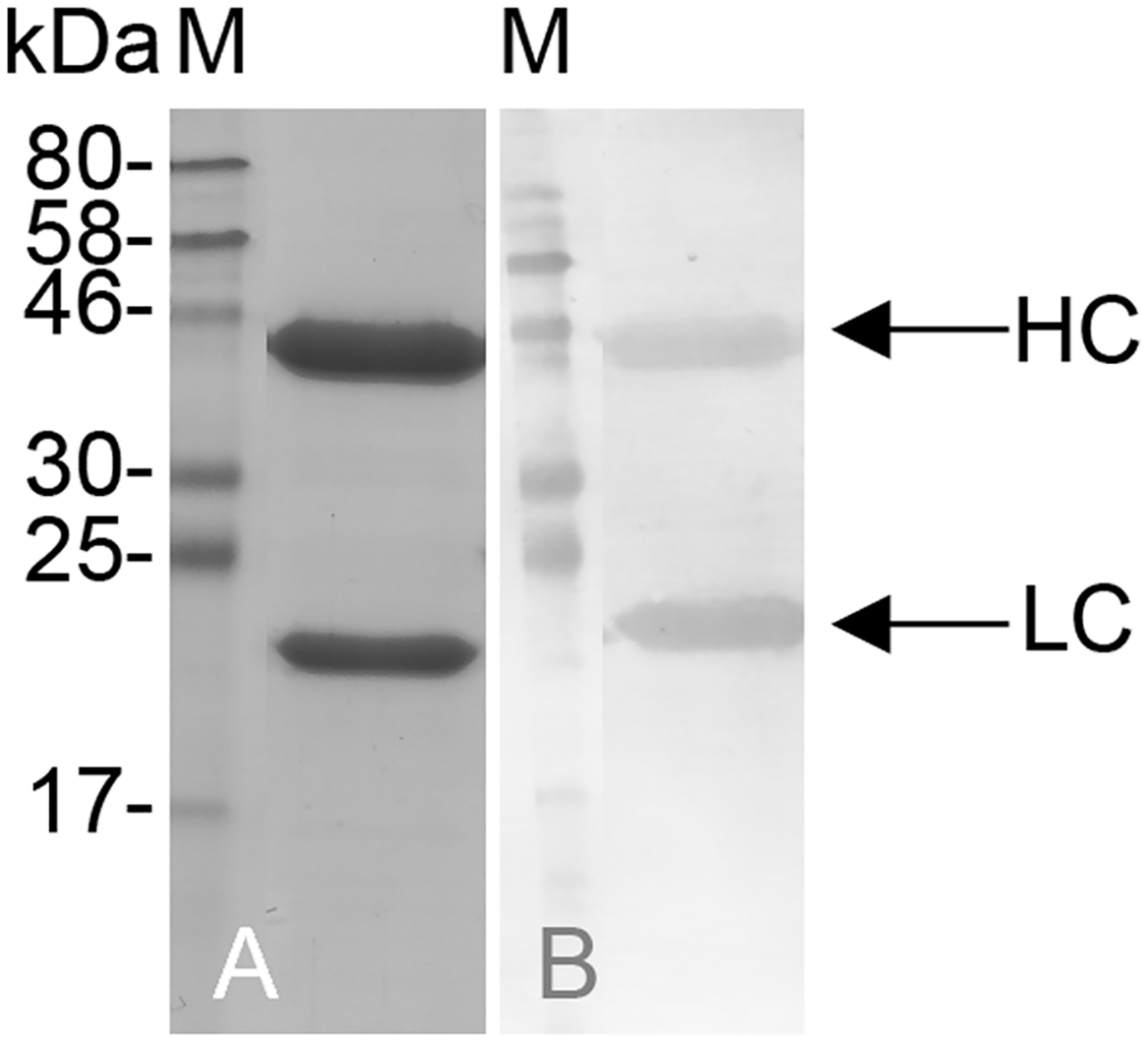
Coomassie-stained SDS-PAGE gel and immunoblot of purified mAb CyDI-4. The purified mAb CyDI-4 (10 μl) was analysed by staining SDS-PAGE gels with Coomassie Brilliant Blue (**A**) and by immunoblot (**B**) stained with an alkaline phosphatase-conjugated polyclonal α-goat mouse antibody (0.12 μg/ml, Dianova 115-055-003) specific for the heavy (**HC**) and light chain (**LC**), **M**: 7 μl Prestained Protein Marker, Broad Range 7–175 kDa (NEB).

For antibody production, the monoclonal hybridoma cell lines were first adapted to serum-free medium during expansion, and were then transferred to a CELLine bioreactor (Sigma-Aldrich, St Louis, USA). The hybridoma supernatant was harvested twice weekly and pooled for purification. Antibodies were purified by affinity chromatography (Protein G Agarose; KPL, Gaithersburg, USA). Neutralised protein samples were analysed by SDS-PAGE and immunoblotting. As an example, Fig 3 shows the analysis of 500 ng mAb CyDI-4 (dialysed and pooled fractions from several purifications) in an SDS-PAGE gel stained with Coomassie Brilliant Blue. This shows two bands, corresponding to the anticipated sizes of the antibody heavy and light chains. Purified mAbs CyDI-1 CyDI-2, and CyDI-4 were analysed in more detail by SPR spectroscopy. The mAbs CyDI-3 and CyDI-12 were excluded from further experiments due to the high dissociation rate of CyDI-3 and problems with antibody expression levels.

### SPR-based analysis of purified monoclonal hCC-specific antibodies

**Fig 4:**
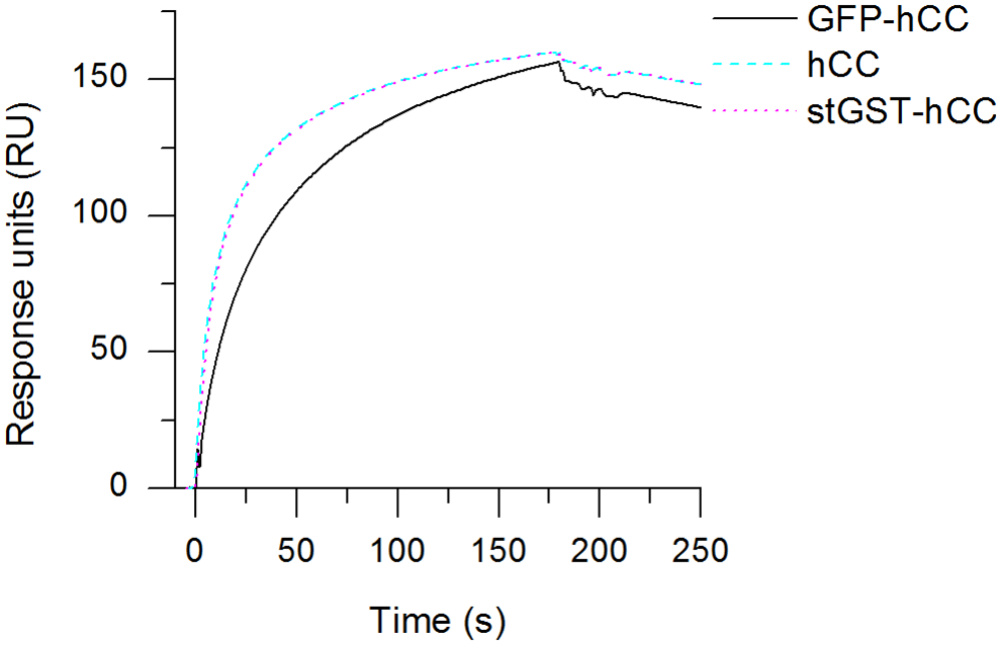
SPR analysis of mAb CyDI-4 based on its ability to bind and release hCC and hCC fusion proteins. Purified mAb CyDI-4 was captured to a response of ~150 RU and hCC/hCC fusion proteins were applied at a concentration of 10 μg/ml for 180 s followed by dissociation. The three tested proteins are shown in different colours.

The detection principle of the envisaged *in vivo* device requires an antibody with similar binding and dissociation properties towards hCC and hCC-fusion proteins. We therefore captured the purified mAbs CyDI-1, CyDI-2 and CyDI-4 until a change in response of ~150 RU was achieved, and tested them against three antigens: hCC as used above, but also GFP-hCC and stGST-hCC (10 μg/ml in each case). Only mAb CyDI-4 showed similar binding and release properties in the presence of hCC and the hCC-fusion proteins (Fig 4). The curves showing the responses for hCC and stGST-hCC coincide precisely, and moderate binding and dissociation was confirmed for each of the tested proteins (albeit marginally slower for stGST-hCC). None of the proteins yielded a signal plateau under these experimental conditions. SPR spectroscopy indicated that the combination of hCC and stGST-hCC generated the most promising results, although another combination was used for further analysis. It is not possible to differentiate between incumbent and replacement proteins using standard immunological assays unless one of the proteins is labelled or additional antibodies are included. Therefore, the combination of hCC and the fluorescent protein GFP-hCC was used for replacement assays on mAb CyDI-4.

### Suitability of mAb CyDI-4, hCC and GFP-hCC as determined by fluorescence-based replacement assays

**Fig 5:**
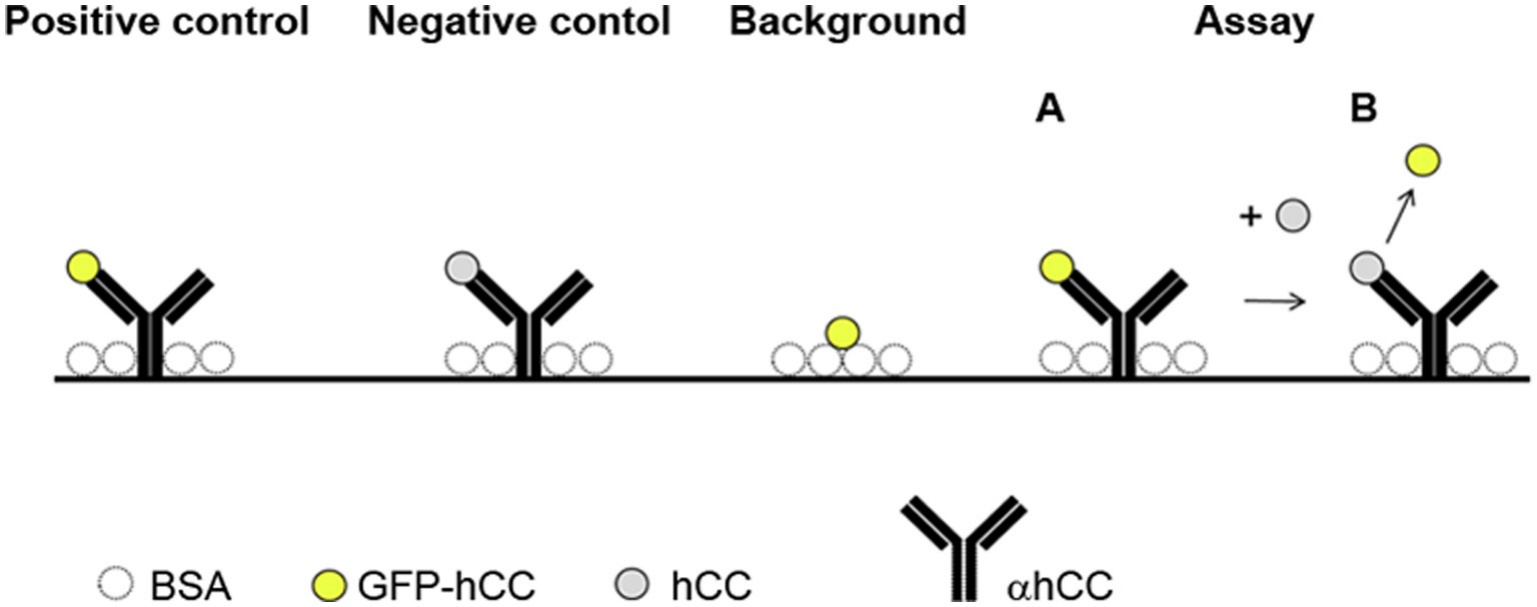
Schematic overview of the fluorescence-based replacement assay and controls (one round of replacement). The hCC-specific antibody was used to bind the fluorescent protein GFP-hCC (positive control) and the non-fluorescent protein hCC (negative control). Background fluorescence was estimated by adding GFP-hCC to a blocked well. One round of replacement occurred when GFP-hCC was captured by mAb CyDI-4 first (**A**) and was then replaced with hCC (**B**).

A fluorescence-based replacement assay was established to determine the ability of hCC to replace the hCC-fusion protein on the monoclonal hCC-specific antibody and vice versa (Fig 5). The assay was carried out in triplicate using black ELISA plates coated with mAb CyDI-4 and blocked with bovine serum albumin (BSA). We used the fluorescent protein GFP-hCC as a positive control and hCC as a negative control, each bound to mAb CyDI-4. GFP-hCC was added to a blocked well to establish the background fluorescence, which was subtracted from the readings taken from all other wells before the signals were normalised against the positive control. The normalised fluorescence intensity was displayed as a percentage relative to the positive control, which was set at 100%.

### Repetitive replacement assay

**Fig 6:**
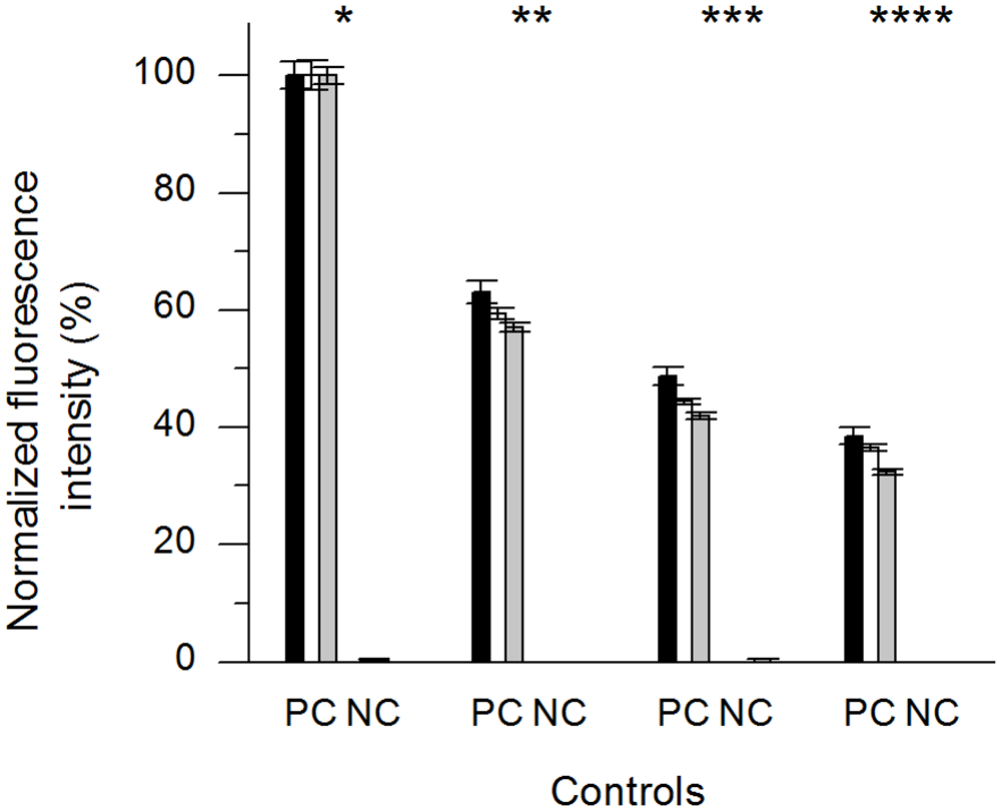
Controls for the repetitive replacement assay. As a positive control in the repetitive replacement assay, GFP-hCC was incubated at the same time as the second antigen in the first cycle of replacement and PBS only was used for the second to fourth cycles. We used hCC as the negative control. Four readouts were obtained, one after each replacement cycle. Each assay was performed in triplicate (error bars represent standard deviations). The background was subtracted from mean values and the resulting fluorescence was normalised to the positive control from the first readout and was plotted as a percentage. The assay was carried out three times (n = 1–3 indicated as black, white and grey bars). **PC:** CyDI-4/BSA/GFP-hCC, **NC:** CyDI-4/BSA/hCC, Asterisks indicate the first to fourth readouts.

**Fig 7:**
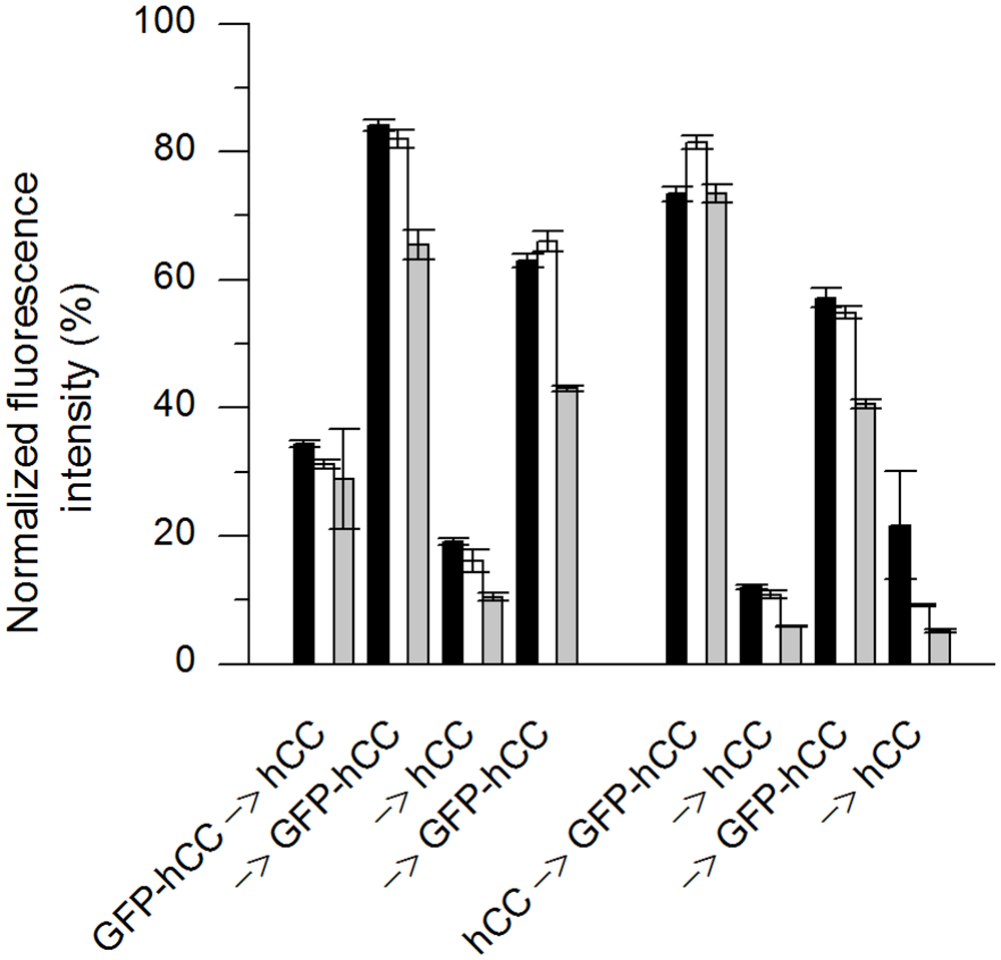
Repetitive replacement assay on mAb CyDI-4 using GFP-hCC and hCC. The repetitive replacement assay was carried out using GFP-hCC and hCC in both directions. The fluorescence intensity was read out after each replacement, and four times in total. The replacement antigens are indicated with an arrow (). Each assay was carried out in triplicate (error bars represent standard deviations). The background was subtracted from mean values and the resulting fluorescence was normalised to the positive control and was plotted as a percentage. The assay was carried out three times (n = 1–3 indicated as black, white and grey bars).

The repetitive replacement assay was used to determine whether four successive cycles of replacement could be achieved with readouts taken after each round. We used hCC and GFP-hCC as the second antigen, but PBS instead of the first antigen in the control wells (Fig 6). Each assay was carried out three times. The fluorescence in the positive control wells declined in a non-linear manner between first and forth readouts, and the fluorescence in the negative control wells varied within the range of background values. The repetitive replacement assays with GFP-hCC and hCC (and vice versa) showed stepwise changes in fluorescence intensity, with the direction of change depending on the replacement protein (Fig 7). The overall reduction in fluorescence intensity between the first and fourth readouts in each experiment was similar to the positive control, and the fluorescence intensity declined with each repetition. We therefore investigated the replacement behaviour of the antibody over time using a time-dependent replacement assay.

### Time-dependent replacement assay

**Fig 8:**
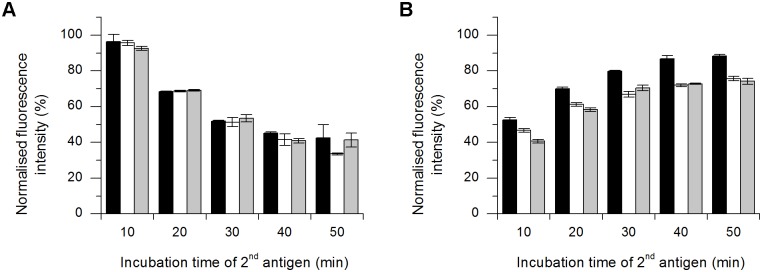
Time-dependent replacement of GFP-hCC with hCC and vice versa. PBS was exchanged for hCC (**A**) and for GFP-hCC (**B**) at 10-min intervals and each assay was carried out in triplicate (error bars represent standard deviations). The background was subtracted from mean values and the resulting fluorescence was normalised to the positive control and was plotted as a percentage. The assay was carried out three times (n = 1–3 indicated as black, white and grey bars).

**Fig 9:**
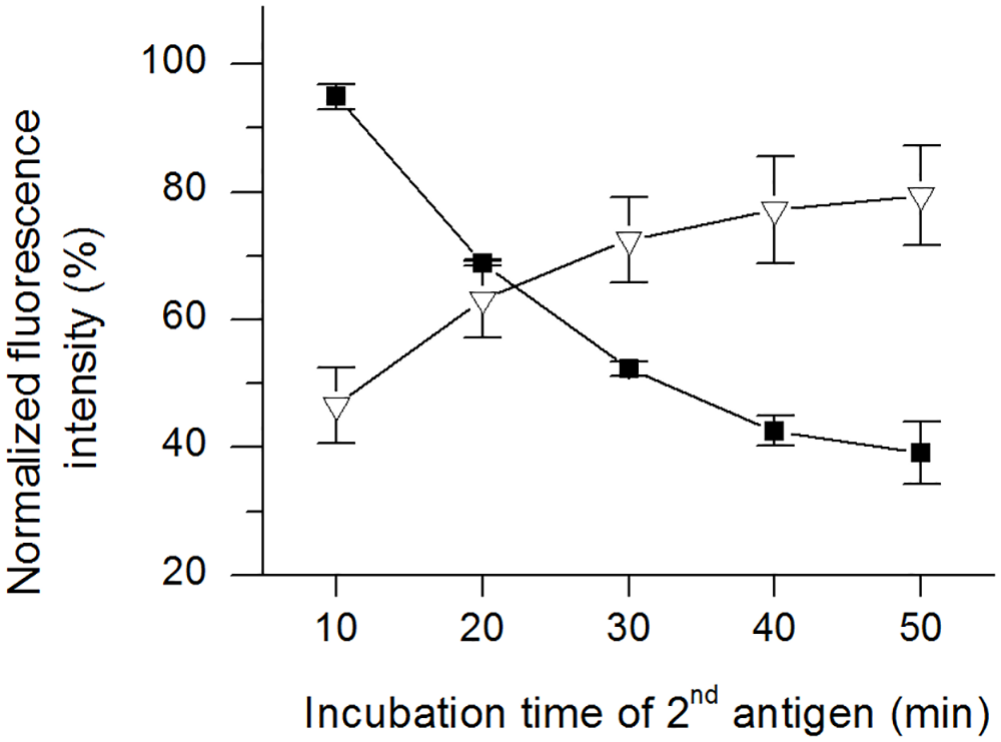
Dynamic range of time-dependent replacement in both directions. The assays were carried out three times. The mean intensity, resulting from assay repetition, was normalised to the positive control and shown as a percentage for the different time points. **Black squares =** GFP-hCC→hCC, **white triangles =** hCC→GFP-hCC.

The time-dependent replacement assay was used to determine the time required for one replacement and was carried out with GFP-hCC and hCC in both directions. In contrast to the repetitive replacement assay, PBS was added to all wells prior to replacement. The PBS was exchanged for the replacement reagent at 10-min intervals. The normalised fluorescence intensities for GFP-hCC replaced by hCC are shown in Fig 8A, and those for the reverse replacement are shown in Fig 8B. When hCC was the replacement reagent, the signal declined in a non-linear manner, and most of the change in signal intensity occurred after 30 min. After 50 min, the mean fluorescence intensity had fallen to 39% of the positive control value (Fig 8A). When GFP-hCC was the replacement reagent, the signal increased in a non-linear manner, and most of the change in signal intensity occurred after 20 min. After 50 min, the mean fluorescence intensity was 79% of the positive control value (Fig 8B). The dynamic range of the time-dependent replacement assay resulting from the repetition of the assay at different time points is presented in Fig 9, which shows the mean intensity values as percentages.

### Replacement assay using reference material

**Fig 10:**
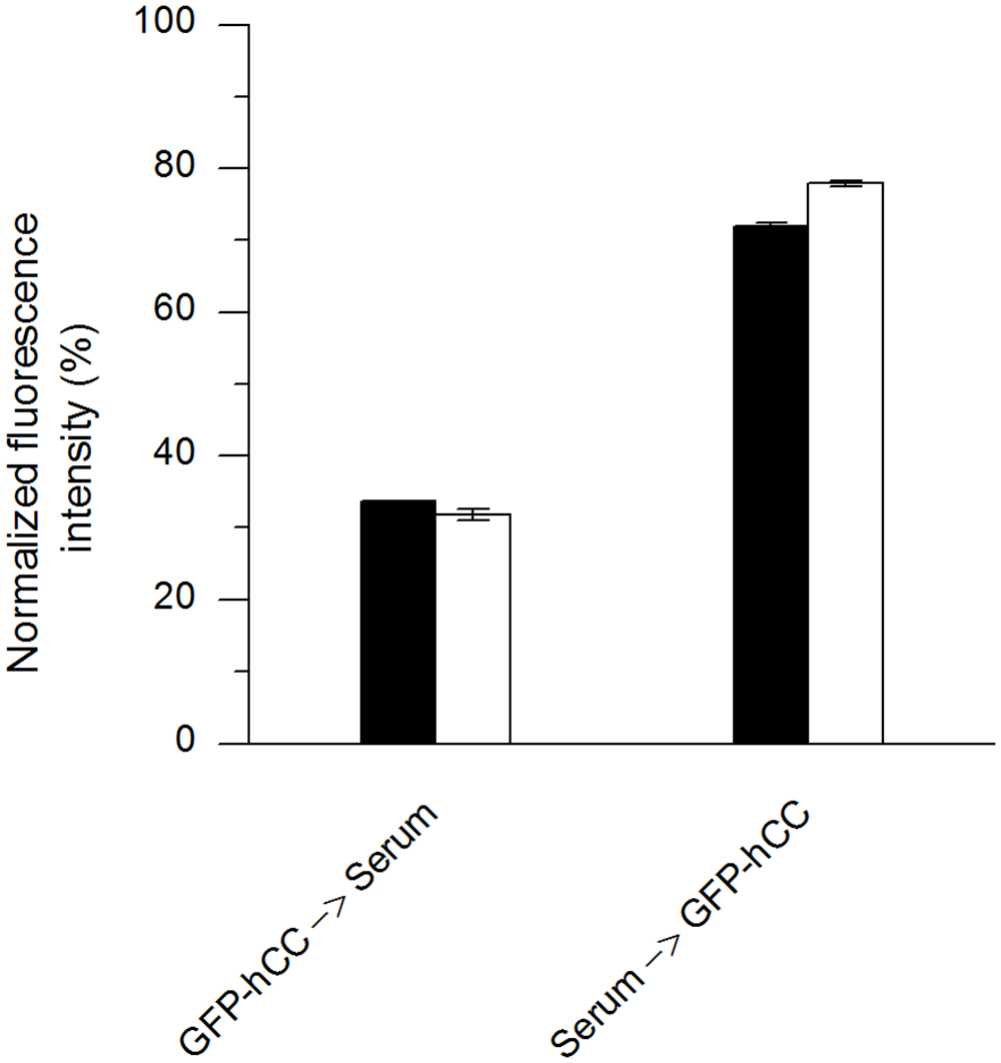
Replacement assay in both directions using reference material. The assays were carried out three times. The mean intensity, resulting from assay repetition, was normalised to the positive control and shown as a percentage for the different time points. **GFP-hCC→Serum:** GFP-hCC replaced with non-diluted reference material, **Serum→GFP-hCC:** non-diluted reference material replaced with GFP-hCC.

All the assays described thus far were based on the use of recombinant hCC in PBS, but the envisaged *in vivo* assay will need to work in the presence of native hCC in serum. We therefore carried out tests using human reference material (ERM-DA471/IFCC (IRMM)) as the replacement reagent, comprising human serum spiked with a defined amount of hCC. A preliminary assay was carried out to determine whether the assay was directly compatible with undiluted human serum (Fig 10). The fluorescence intensity was 33% and 75% of the positive control value respectively, after replacement in both directions.

The second assay with reference material was used to determine whether the range of the assay is compatible with the physiological and pathological concentrations of hCC in serum. We therefore prepared hCC-depleted normal human serum (dNHuS) and confirmed the success of depletion using a sandwich ELISA (sELISA). The dNHuS absorption signal was similar to the background, whereas that of the non-depleted serum was as high as the positive control (data not shown). The detection limit of the sELISA was 4 ng/ml hCC in PBS (data not shown) confirming that the hCC content of dNHuS was ≤ 4 ng/ml.

**Fig 11:**
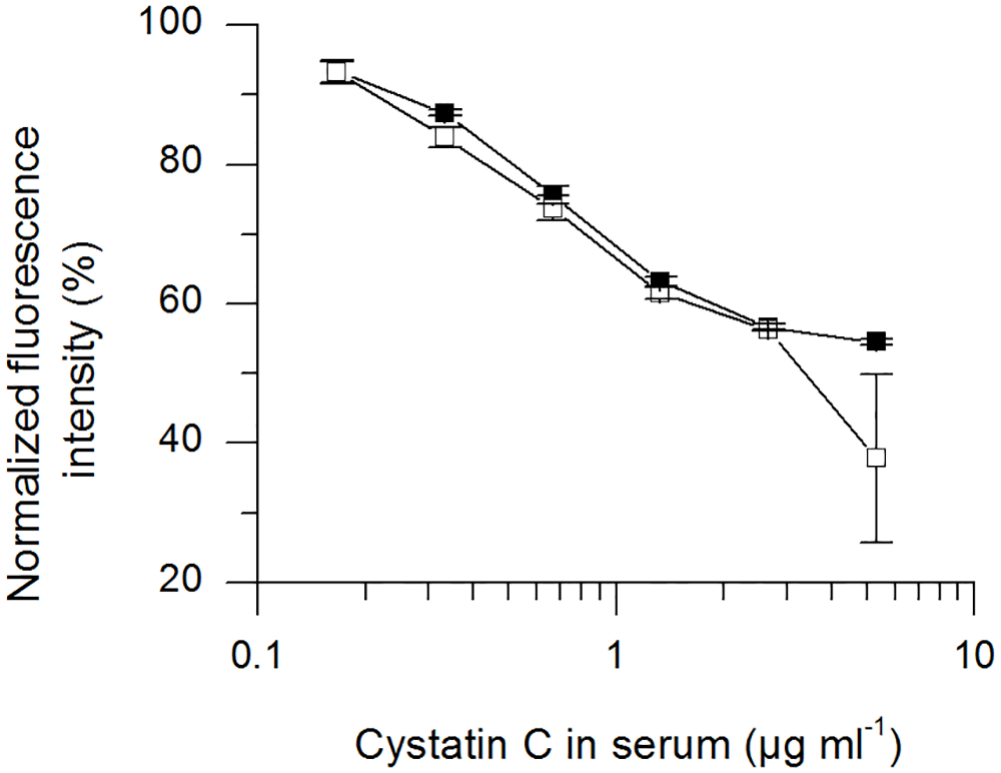
Replacement assay with serially-diluted reference material. Serial dilutions of reference material in dNHuS were used to replace GFP-hCC. The hCC concentration in serum (μg/ml) is plotted against the fluorescence readout. A logarithmic scale was used on the x-axis. The assay was carried out twice, represented by black circles and white triangles.

The reference material was serially diluted with dNHuS and used as a replacement reagent for GFP-hCC to determine the range of the replacement assay. We observed a concentration-dependent reduction in fluorescence intensity (Fig 11), although an exact relationship between hCC concentration and fluorescence intensity could not be determined in part because only one repetition could be conducted.

## Discussion

High serum concentrations of hCC correlating with certain kidney diseases can indicate an increased risk of cardiovascular disease [18,19] and other disorders [12,22,23]. The *in vivo* measurement of hCC levels would therefore allow long-term remote monitoring and could thus determine the progression of various diseases. However, all validated hCC assays are currently based on *ex vivo* detection. As a first step towards the development of *in vivo* hCC assays based on antibodies and recombinant marker proteins that can be integrated with indwelling medical devices, we produced recombinant hCC, GST and three hCC-fusion proteins using *E*. *coli* BL21 (DE3) and HEK239T cells accordingly. The integrity of the purified proteins was confirmed by SDS-PAGE and immunoblotting, which showed that the observed bands matched the theoretical protein sizes predicted by ExPASy ProtParam (http://web.expasy.org/protparam/). The immunogenicity of hCC in mice was predicted using the Immune Epitope database and Analysis research tool to identify potential MHC-II T-cell epitopes [24], which indicated moderate to low affinities. Even though such T-cell epitopes can be sufficient for the induction of a specific immune response [25], hCC purified by IMAC and AEC did not induce a specific immune response in the mice used in our experiments. This result was unexpected because murine monoclonal hCC-specific antibodies are commercially available (Hytest). These antibodies have been generated using urinary-hCC from patients with renal impairment [26]. Purified urinary hCC is a mixture of three variants, one full-length polypeptide with an isoelectric point (pI) of 9.2, and two N-terminal truncations (of eight and nine amino acids, respectively) with pI values of 7.8 [27], whereas our recombinant full-length hCC was purified by AEC thus yielding a single polypeptide with a specific pI value. Another explanation may reflect possible differences in the immunisation procedure (injection route, regimen and adjuvant) because the mouse strain was the same in both studies. We therefore used stGST-hCC to generate hCC-specific antibodies in mice. Thereby we achieved titres in the range 1:51 200 to 1:102 400 prior to fusion. We generated hybridomas and subjected them to limiting dilution to establish monoclonal hybridoma cell lines, 12 of which were positive for hCC binding when tested by ELISA and sELISA. We found that sELISA was necessary to exclude antibodies that bind hCC in ELISA experiments but not when tested by SPR spectroscopy, an effect that is most probably caused by the complete or partial denaturation of proteins induced by direct coating to the polystyrene surface, as observed for other antibodies generated in house (data not published).

Because we sought to develop a bioassay suitable for repetitive measurements with the same set of antibodies without stripping, we were particularly interested in the antibody dissociation characteristics. SPR spectroscopy is used extensively to analyse protein interactions [28–31] but to our knowledge, this is the first report describing the characteristics of hCC-specific antibodies based on SPR spectroscopy. We excluded all antibodies with a response greater than 180 RU for antibody capture but lower than 50 RU for hCC binding, reflecting their low binding affinity. Such low binding affinities may reflect an unsuitable paratope structure or low activity after purification (which is indicative of an inherently unstable antibody), neither of which would be useful for the development of an *in vivo* detection system. We also excluded antibodies showing identical binding characteristics because these were likely to represent the same antibody polypeptide. Ultimately, we found that 5 of the 12 potentially different mAbs showed suitable yet distinct hCC association and dissociation characteristics (Fig 2). CyDI-1 was characterised by a very slow off-rate and thereby almost no dissociation, suggesting it would be useful as a common detection antibody. This was verified by SPR spectroscopy, revealing a dissociation constant of 2.9 x 10^−10^ M which is close to the dissociation constants reported for high-affinity antibodies against H5N1 *Influenzavirus A* strains [32]. The fastest dissociation was observed for mAb CyDI-3, followed by mAbs CyDI-2, CyDI-4 and CyDI-12. We also looked for antibodies with similar association and dissociation characteristics against hCC and hCC-fusion proteins, because this property is essential for assays based on replacement. The mAbs CyDI-1, CyDI-2 and CyDI-4 were analysed by SPR spectroscopy using hCC, stGST-hCC and GFP-hCC as the antigens (data not shown for mAbs CyDI-1 or CyDI-2). Only mAb CyDI-4 showed the same association and dissociation characteristics towards hCC and stGST-hCC by SPR spectroscopy, and slightly lower affinity for GFP-hCC (Fig 4). These SPR data identified mAb CyDI-4 as potentially the most suitable for replacement studies. Although the SPR spectroscopy experiments indicated that hCC and stGST-hCC were best suited for replacement studies, another combination was required for further analysis. We did not wish to differentiate between stGST-hCC and hCC by protein modification (e.g. labelling) or the use of additional antibodies, because the former may alter the binding affinity and the latter will not be used in the end application. Hence, we used the combination of hCC and the fluorescent protein GFP-hCC to analyse replacement on mAb CyDI-4.

In the repetitive replacement assay, the negative control yielded a lower fluorescent signal than the established background fluorescence, and there was a non-linear decline in fluorescence intensity from the first to fourth measurements in the positive control (Fig 6). This phenomenon was probably caused by the dissociation of GFP-hCC from the antibody. The effect of bleaching was not detectable over five measurements and we therefore concluded this did not contribute to the decline in signal intensity. Repetitive replacement was confirmed by our repetitive replacement assay (Fig 7), which showed a stepwise decline in fluorescence intensity depending on the replacement antigen. With two exceptions, the standard deviation for triplicates was below 2.4% in 91.6% of cases, and was even below 1% in 50% of cases. Comparing the overall normalised fluorescence intensity across assays, we observed a higher standard deviation (6.5–11.3% in 50% of the cases and 1.4–4% in the others). Although we observed stepwise changes in fluorescence intensity, the maximum fluorescence intensity differed when comparing the readouts from the first and third or the second and fourth replacements, where an overall reduction in fluorescence intensity was observed. The fluorescence measurements after replacement were above background and below the positive control value, suggesting that replacement was incomplete. This may reflect the different starting conditions in the second replacement step, and would have continued in the subsequent replacements. The decline in fluorescence intensity indicated a higher affinity for hCC. This was not unexpected because it was predicted by SPR analysis with mAb CyDI-4 and different antigens, i.e. the antibody bound to GFP-hCC with marginally lower affinity than hCC and stGST-hCC.

In addition to the repeatability of replacement, we investigated the time-dependent change in fluorescence during one round of replacement to determine whether the time needed for replacement was compatible with an implant. The time-dependent replacement assay showed a non-linear decrease and increase in the signal intensity, with only small overall changes at the end of incubation (Figs 8 and 9). Most of the change in fluorescence intensity took place within the first 20–30 min, which is fast for this type of mechanism. Antibodies with slower dissociation rates would need more time, making them unsuitable for this approach. Faster dissociation as seen for mAb CyDI-3 in the SPR spectroscopy experiments could not be monitored in the replacement assay because the signal fell to near background after replacement (data not shown). It is likely that the rapid dissociation caused the loss of signal during the washing steps. Normally, the hCC concentration in serum varies during the day [33]. Therefore a quicker assay that enables complete hCC surveillance throughput an entire day is unnecessary, confirming that our assay fulfils all time-based requirements. Regarding the dynamics of time-dependent replacement, the increase in fluorescence observed with GFP-hCC was more limited than the reduction in fluorescence observed with hCC. This supports our hypothesis, as suggested by the repetitive replacement assay, that a slightly higher affinity towards hCC might cause such a difference.

The use of reference material was necessary to determine the impact of other proteins in the serum on the sensitivity of the assay for hCC. Most hCC ELISAs use diluted serum samples [34–36]. However, the replacement assay with reference material produced similar results to the assay in which hCC was presented as a pure substance dissolved in PBS, and the standard deviations for triplicates were smaller (all below 0.8%). As for the other replacement assays, the standard deviations for assay repetitions were higher than those observed for triplicates (1.3% and 4.3%, respectively) but lower than observed for replacement assays using hCC in PBS. This reflects the ability of serum proteins to block the assay.

Finally, we investigated the assay range and specifically whether it covered typical physiological and pathological parameters for the concentration of hCC in serum. The physiological range of serum hCC concentrations including both genders is 0.51–0.98 mg/l [37], whereas pathological serum hCC concentrations in the elderly can increase to 6.11 mg/l in chronic kidney disease [38]. The assay range covered the physiological range of human hCC concentrations in serum, and also most of the pathological range. With one exception, the standard deviation for assay repetition was below 2.4%, confirming excellent reproducibility. However, the assay could only be carried out twice so it was not possible to determine the statistical significance of these data. Although a dose–response relationship was observed, it was not possible to calculate a precise relationship between hCC concentration and the fluorescence signal.

In summary, state-of-the-art immunoassays are generally limited to *in vitro* and to non-repetitive use. This study demonstrates that a replacement assay may be a feasible option for the continuous measurement of serum hCC levels. Continuous measurement in return is the first step allowing immunoassays to be integrated into implantable biosensors and *in vivo* measuring systems. We found that mAb CyDI-4 was an excellent candidate antibody for the replacement assay, although the combination of GFP-hCC and hCC was not ideal and was only used to confirm the detection requirements. Although a replacement-based bioassay is possible in principle, further optimisation would be necessary, e.g. by engineering the antibody and/or the fusion protein to improve their binding and release characteristics.

## Materials and Methods

### Ethics statement

The animal experiments were officially approved by the Landesamt für Natur, Umwelt und Verbraucherschutz Nordrhein-Westfalen (LANUV), reference number 84–02.05.30.12.097. All animals received humane care in accordance with the requirements of the German Tierschutzgesetz, §8 Abs. 1 and in accordance with the Guide for the Care and Use of Laboratory Animals published by the National Institutes of Health.

### Material

For protein production and purification we used a Glutathione HiCap Matrix (Qiagen), Ni-NTA Superflow Cartridges (Qiagen), Protein A Ceramic HyperD^®^ F (Pall), Q Ceramic HyperD^®^ F (Pall), a CELLine bioreactor CL 350 (Sigma-Aldrich) and Protein G Agarose (KPL). We used a goat α-mouse IgG (Fcγ) secondary antibody labelled with horseradish peroxidase purchased from Sigma-Aldrich, goat α-human IgG from Thermo Fisher Scientific. Norman human serum (S1-100ML) was acquired from EMD Millipore (Billerica, USA) and Biotin LC hydrazide was obtained from Santa Cruz Biotechnology (Dallas, USA). Reference material ERM-DA471/IFCC was acquired from the Institute for Reference Materials and Measurements, Belgium.

### Bacteria and cell lines

We used *E*. *coli* strain DH5α for the amplification of plasmid DNA and strain BL21 (DE3) for protein expression (Novagen, Merck KGaA, Darmstadt, Germany). Human embryonic kidney cell line 293, established by transfection with “sheared DNA of adenovirus type 5” (HEK293T, ACC 635, Leibniz Institute DSMZ-German Collection of microorganisms and Cell cultures, DSMZ) was used for protein production, and cell line SP2/0-AG14 (ACC 146, DSMZ) was used to generate hybridoma lines.

### Cloning for the production of hCC, hCC fusion proteins and GST

The hCC amino acid sequence (GenBank CAA36497.1) was randomly reverse translated using CLC Main Workbench v6.9.1 (CLC bio, Aarhus, Denmark) and the resulting sequence was codon optimised for *E*. *coli* and *H*. *sapiens* (http://gcua.schoedl.de/, http://www.kazusa.or.jp/codon/). The synthetic gene was prepared by Eurofins MWG Operon. The hCC insert was prepared for cloning by SOE-PCR [39] and standard PCR [40]. The inserts for hCC and the hCC fusion proteins were transferred into pMS-based vectors for expression in HEK293T cells [41] using pMS-LH22-IV, pMS-EGFRex-Hinge-Fc and pMS-L-GFP-scFvEpCAM as the source plasmids (kindly provided by Katharina Kolberg, Dr. Christoph Stein, Dr. Christiane Püttmann and Lea Hain, Institute for Applied Medical Engineering, Aachen). A hCC fusion protein was produced in *E*. *coli* BL21 (DE3) cells by transferring the insert into pGEX5x3-based vectors using pGEX5x3-E2 (1b) as the source plasmid. The pGEX5x3-E2 (1b) vector was kindly provided by Dr. Püttmann (Institute for Applied Medical Engineering, Aachen). All primers, cloning steps and restriction enzymes are summarised in S1–S4 Tables. All restriction enzymes were provided by New England Biolabs (Ipswich, USA). All cloning products were verified by sequencing.

### Production and purification of proteins in bacteria

*E*. *coli* BL21 (DE3) cells were transformed by heat shock and a pre-culture was cultivated by shaking at 37°C, overnight. The main culture (4 x 400 ml in 2-L flasks) was inoculated with the pre-culture at a ratio of 1:50 and incubated as above until the OD_600_ reached 0.8–1. At this point, protein expression was induced with isopropyl β-D-1-thiogalactopyranoside (0.1 mM final concentration). The cells were harvested after 4 h and centrifuged at 4000 g for 10 min then stored at –80°C. The stGST-hCC protein was purified as described in the “Manual Purification Protocol” from the “Glutathione Affinity Handbook, May 2010” (Qiagen).

### Production and purification of proteins in eukaryotic cells

HEK293T cells (~70–80% adherent) were seeded in a six-well plate. We mixed 300 μl RPMI medium (Thermo Fisher Scientific), 2–3 μg DNA and 2–3 μl Roti^®^-Fect (Carl Roth) by gentle inversion, incubated the mixture for 30 min at room temperature, and added it dropwise to the cells followed by incubation at 37°C, 5% CO_2_ for 3–4 h before adding R10 culture medium (10% (v/v) foetal calf serum (FCS) in RPMI).

Selection pressure was applied 2–3 d later by adding 100 μg/ml Zeocin^™^, and the cells were selected on the basis of resistance to the antibiotic for 2 weeks. The cells were diluted ~1:10 twice weekly, and after 2 weeks the products were analysed by SDS-PAGE [42] and immunoblot [43]. The cells were then expanded to one triple flask (Nunclon™Δ) and the supernatant was harvested three times per week. The cells were removed prior to collection (500 g, 25 min, 4°C) and the pooled supernatant fractions were stored at 4°C ready for protein purification.

The hCC and GFP-hCC proteins were purified by IMAC [44]. The cell supernatant was mixed with 10x IMAC-BW buffer (500 mM Na_2_HPO_4_, 3 M NaCl and 100 mM imidazole (pH 8.0)) and forced through glass and cellulose acetate filters at the same time (0.45 μm, Sartorius) using compressed air. The protein was then loaded onto a Ni-NTA Superflow Cartridge on an ÄKTA purifier and eluted using a gradient of IMAC elution buffer (50 mM Na_2_HPO_4_, 300 mM NaCl, 250 mM imidazole, pH 8.0). Batch processes were also carried out, this time using step elution. The eluted hCC protein was dialysed against AEC buffer (20 mM ethanolamine, 30 mM NaCl, pH 9.0) and purified by gravity flow-through chromatography with AEC Q Ceramic HyperD F resin (Pall).

The hCC-Hinge-Fc protein was purified by Protein A affinity chromatography after mixing with 4x Protein A binding buffer (1 M Tris, pH 8.0), filtering as above and passing through a column containing Protein A Ceramic HyperD F on an ÄKTA purifier. The column was washed with 1x Protein A buffer and the product was eluted in Protein A elution buffer (0.2 M trisodium citrate, pH 2.5). The eluted protein fraction was neutralised with Protein A neutralisation buffer (1 M Tris, pH 9.0). The analytical purification of antibodies was carried out in batch mode. Samples were analysed by SDS-PAGE [42] and immunoblot [43].

### Immunisation of mice and measurement of titres

Mice were immunised essentially as previously described [25]. Briefly, 60 μg of stGST-hCC was injected subcutaneously as the prime immunisation, followed later by a 30-μg boost. Mice were boosted on three consecutive days without adjuvant prior to the generation of hybridoma lines.

Titres were determined as previously described [25] with the protocol adapted for antigen coating, the dilution range and the secondary antibody. We coated wells with 50 ng stGST-hCC/hCC or GST per well (1 μg/ml) dissolved in 50% (v/v) PBS and 50% (v/v) 2x coating buffer (0.2 M Na_2_CO_3_, 0.2 M NaHCO_3_, pH 9.6). The primary antibody was serially diluted in the range 1:800 to 1:4 096 000.

### Hybridoma generation and limiting dilution

B-cells were immortalised by creating hybridoma lines [45] using murine myeloma cell line SP2/0-AG14 (DSMZ) in a 1:1–1:2 ratio. The cells were fused by exposing them to polyethylene glycol prior to selection in HAT medium (1x hypoxanthine-aminopterin-thymidine, 0.5 μg/ml IL-6 (Pan Biotech, Aidenbach, Germany), 20% (v/v) FCS (Biochrom, Cambridge, UK), 1x β-mercaptoethanol in RPMI). Cells were selected for 2 weeks in 96-well plates. Positively selected cells were subjected to limiting dilution in HT-medium (1x hypoxanthine-thymidine (Pan-Biotech) in RPMI). Monoclonal hybridoma cell lines were adapted stepwise to R10 culture medium.

### Selection of monoclonal hybridoma cell lines

Monoclonal hybridoma cell lines were selected by indirect ELISA and sELISA [46]. The indirect ELISA (with GST, stGST-hCC, hCC and BSA) was carried out as described above for the measurement of titres except that hybridoma supernatant was used. For sELISA, a goat α-human IgG (Thermo Fisher Scientific) was used to capture hCC-Hinge-Fc before applying the hybridoma supernatant.

### Production and purification of monoclonal antibodies

After the selection of monoclonal cell lines by ELISA and sELISA, the cells were adapted to and expanded in serum-free medium (ISF-1, EMD Millipore), and 8 x 10^6^ cells were transferred into a CELLine bioreactor (Sigma-Aldrich). The supernatant was harvested twice weekly, separated from the majority of cells by centrifugation (200 g, 5 min, room temperature) and stored at 4°C ready for protein purification. The monoclonal hCC-specific antibodies were purified on a Protein G affinity column (KPL) according to the manufacturer’s recommendations. Prior to purification, the supernatant from different harvest time points was pooled and passed through a 0.45-μm PVDF syringe filter to remove all particles. Samples were analysed by SDS-PAGE [42] and immunoblot [43].

### SPR-based analysis of antibodies from monoclonal hybridoma supernatants

For kinetic analysis, mAbs from pre-diluted supernatants from hybridoma cultures (1:6 in PBS) were initially captured on a RAM-Fc chip for 180 s and Protein G-purified mAbs after expression by monoclonal hybridoma cell lines were initially captured to a response of 150 RU. Recombinant hCC (18 μg/ml for the analysis of hybridoma supernatants and 10 μg/ml for the analysis of purified monoclonal antibodies, respectively) was injected at a flow rate of 30 μl/min for 180 s. Dissociation was followed for 180 s. Between measurements, the surface was regenerated by pulsing for 1 min with 30 mM HCl. Buffer injections were used for double referencing. Binding curves were evaluated using BiaEval Software (GE Healthcare).

### Depletion of hCC from normal human serum

Normal human serum was depleted for hCC (dNHuS) and used to dilute the reference material in the replacement assays. Depletion was achieved using an affinity matrix prepared from mAb CyDI-1 and NHS-activated Sepharose^™^ 4 Fast Flow (GE Healthcare). A Vivaspin 15R column (30 MWCO; Sartorius-Stedim, Göttingen, Germany) was used to concentrate 6 mg of the antibody and exchange the buffer.

Antibodies were coupled to 0.5 ml NHS-activated Sepharose according to the manufacturer’s recommendations for 1 h at room temperature. After overnight inactivation, the matrix was washed with NHS buffer C (0.1 M Tris-HCl, pH 8.5) and NHS buffer D (0.1 M acetate, pH 4) alternating six times. Prior to storage, we carried out one elution step with sterile glycine buffer (0.2 M glycine, pH 2.9) and a neutralisation step with sterile PBS.

For the depletion of 5 ml normal human serum, we carried out two incubation steps using the mAb CyDI-1 matrix, first using a batch approach (overhead mixing, 15 min, room temperature), followed by a gravity flow approach at room temperature. An intermediate elution step was carried out as described above.

### Confirmation of hCC depletion by sELISA

Successful depletion was confirmed by sELISA using mAb CyDI-1 as the capture antibody and a biotinylated mAb CyDI-2 as the secondary antibody. For the biotinylation of mAb CyDI-2, 1.5 mg CyDI-2 in 2 ml of cold buffer A (0.1 M sodium acetate, pH 5.5) was incubated with 756 μl buffer B (20 mM sodium metaperiodate) for 30 min at 4°C in the dark. The solution was dialysed against 1x PBS (overnight at 4°C) and biotinylation buffer C was added to the protein solution at a 1:9 v/v ratio followed by end-over-end mixing for 2 h at room temperature. The solution was dialysed as above. We then coated a polypropylene microwell plate (Ratiolab GmbH, Dreieich, Germany) with 500 ng mAb CyDI-1 dissolved in 50% (v/v) PBS and 50% (v/v) 2x coating buffer (0.2 M Na_2_CO_3_, 0.2 M NaHCO_3_, pH 9.6). We added 300 μl of 5% milk powder in PBS as a blocking agent, then analysed 50 μl dNHuS and non-dNHuS. We added 50 μl biotinylated-mAb CyDI-2 and peroxidase-conjugated streptavidin (1:5000 in PBS) (Jackson ImmunoResearch Laboratories, West Grove, USA) and carried out detection as described above.

### Fluorescence-based replacement assays

Antigen replacement on mAb CyDI-4 was visualised using the fluorescent fusion protein GFP-hCC in addition to hCC. The assays were carried out using black high-binding plates (Greiner) in triplicates at room temperature. All steps involved 50-μl aliquots except the blocking step, in which we applied 300 μl of blocking buffer. The coating solution comprised the protein dissolved in 50% (v/v) PBS and 50% (v/v) 2x coating buffer (0.2 M Na_2_CO_3_, 0.2 M NaHCO_3_, pH 9.6). All other solutions comprised the protein in PBS only. Between steps, the plates were emptied and washed three times with PBST. The steps are summarised in Table 1. Only one antigen at a time was used and the order of application was varied so we could test both directions of replacement. A Tecan GENios Pro System (Tecan, Männedorf, Switzerland) was used for readouts after filling the wells with PBS (485 nm excitation, 520 nm emission, circle pattern, 3 x 3 number of reads, gain 50, 10 s orbital shaking at 100 rpm and 20 s to settle prior to measurement, 37°C).

**Table 1:** Fluorescence-based replacement assay steps.

Step	Component	Concentration	Volume	Incubation temperature/time
Coating antibody	CyDI-4	15 nM	50 μl	4°C, overnight
Blocking	BSA	1% (w/v)	300 μl	1 h
First antigen	GFP-hCC/hCC or reference serum	300.8 nM for GFP-hCC/hCC	50 μl	1 h
Second antigen	hCC or reference serum/GFP-hCC	300.8 nM for hCC/GFP-hCC	50 μl	1 h
Readout	PBS	1x	50 μl	-

The assay was also carried out using the fluorescent protein as the second antigen (positive control) and the non-fluorescent protein as the second antigen (negative control). PBS was used instead of the first antigen and for incubation steps after the first readout. Background fluorescence was measured using GFP-hCC alone (blocking and first antigen steps, Table 1).

### Repetitive replacement assay

For the repetitive replacement assay, alternating antigen solutions were used for replacement followed by readouts after each step. In total the assay was read out four times. Each assay was carried out three times.

### Time-dependent replacement assay

For the time-dependent replacement assay, PBS was added prior to the second antigen, which was added to three wells at 10-min intervals. The readout of the assay was taken after 1 h. The assay was carried out three times.

### Replacement assays with reference material

Reference material ERM-DA471/IFCC (IRMM) was used for the replacement assays instead of recombinant hCC in PBS. The reference material is human serum standard spiked with a defined amount of hCC. The replacement assay with reference material was carried out in both directions using the procedure described for the repetitive replacement assay until the first measurement, except that non-diluted reference material was used instead of hCC in PBS. As a second replacement assay, the reference material was serially diluted (1:1) with dNHuS and was used to replace GFP-hCC using the procedure described for the repetitive replacement assay until the first measurement. Due to limitations in the available reference material, both assays were performed in triplicate with one repetition in one direction.

## Supporting Information

S1 Table SOE-PCR cloning. Primer combinations and templates used for the three-step SOE-PCR, plus the target plasmids and restriction enzymes.
(DOCX)

S2 Table PCR cloning. Primer combinations and templates used for standard PCR cloning, plus the target plasmids and restriction enzymes.
(DOCX)

S3 Table *In vivo* amplification and cloning. Enzyme combinations and plasmids used for *in vivo* cloning.
(DOCX)

S4 Table Primers. Primer sequences used for cloning.
(DOCX)
