# Supplementary material for: Development of a Competitive Cystatin C-Specific Bioassay Suitable for Repetitive Measurements
Source: PLoS One. 2016 Jan 22;11(1):e0147177. doi: 10.1371/journal.pone.0147177 (PMC4723070; doi:10.1371/journal.pone.0147177)
Supplement: S1 Table — Primer combinations and templates used for the three-step SOE-PCR, plus the target plasmids and restriction enzymes. (DOCX) [file pone.0147177.s001.docx]

S1 Table. SOE-PCR cloning.

Primer combinations and templates used for the three-step SOE-PCR, plus the target plasmids and restriction enzymes.

| Plasmid | PCR step | 5’Primer | 3’ Primer | Template | Restriction enzymes and target plasmid |  |
| --- | --- | --- | --- | --- | --- | --- |
| pMS-L-hCC_2 | R1 | 5’ IgK | 3’ IgK | pMS-LH22-IV | *Nhe*I/*Not*I;  pMS-L-hCC-IV | |
|  | R2 | 5’ IgK-hCC | 3’-HCC | pCR2.1-hCystatin |  |  |
|  | R4 | 5’ IgK | 3’-HCC | R3 |  |  |
| pGEXTF-Ago1 | R1 | 5’ GST | 3’ GST | pGEX5x3-E2(1b) | *Eco*NI/*Not*I*;*  pGEX5x3-E2(1b) | |
|  | R2 | 5’-hCC (GST) | 3’-HCC | pCR2.1-hCystatin |  |  |
|  | R4 | 5’ GST | 3’-HCC | R3 |  |  |
