# Supplementary material for: Development of a Competitive Cystatin C-Specific Bioassay Suitable for Repetitive Measurements
Source: PLoS One. 2016 Jan 22;11(1):e0147177. doi: 10.1371/journal.pone.0147177 (PMC4723070; doi:10.1371/journal.pone.0147177)
Supplement: S2 Table — Primer combinations and templates used for standard PCR cloning, plus the target plasmids and restriction enzymes. (DOCX) [file pone.0147177.s002.docx]

S2 Table. PCR cloning.

Primer combinations and templates used for standard PCR cloning, plus the target plasmids and restriction enzymes.

| Plasmid | 5’-Primer | 3’-Primer | Template | Restriction enzymes and target plasmid |
| --- | --- | --- | --- | --- |
| pMS-nathCC_2 | 5’ NheI natlnt | 3’-HCC | pCR2.1-hCystatin | NheI/NotI;  pMS-L-hCC-IV |
| pMS-L-GFP-hCC-MH | 5`Sfi-hCC | 3`-HCC | pCR2.1-hCystatin | SfiI/NotI;  pMS-L-GFP-scFvEpCAM |
