# Supplementary material for: Development of a Competitive Cystatin C-Specific Bioassay Suitable for Repetitive Measurements
Source: PLoS One. 2016 Jan 22;11(1):e0147177. doi: 10.1371/journal.pone.0147177 (PMC4723070; doi:10.1371/journal.pone.0147177)
Supplement: S3 Table — Enzyme combinations and plasmids used for in vivo cloning. (DOCX) [file pone.0147177.s003.docx]

S3 Table. *In vivo* amplification and cloning.

Enzyme combinations and plasmids used for in vivo cloning.

| Plasmid | Insert | Restriction enzymes and target plasmid |
| --- | --- | --- |
| pMS-L-hCC-Hinge-Fc | pMS-L-hCC_2 | *Nhe*I/*Not*I;  pMS-EGFRex-Hinge-Fc |
| pGEX5x3_MBTF | - | *Nco*I/*Not*I  pGEX5x3-E2(1b) |
