# Supplementary material for: Development of a Competitive Cystatin C-Specific Bioassay Suitable for Repetitive Measurements
Source: PLoS One. 2016 Jan 22;11(1):e0147177. doi: 10.1371/journal.pone.0147177 (PMC4723070; doi:10.1371/journal.pone.0147177)
Supplement: S4 Table — Primer sequences used for cloning. (DOCX) [file pone.0147177.s004.docx]

S4 Table. Primers.

Primer sequences used for cloning.

| Oligonucleotide | Sequence (5’->3’) |
| --- | --- |
| 5’ GST | GGAAACAGTATTCATGTCCCCTATACTAGG |
| 3’ GST | GAGGTTTGCCAGGGCTGGATTTTGGAGGATGGTC |
| 5’ hCC(GST) | GACCATCCTCCAAAATCCAGCCCTGGCAAACCTC |
| 3’ IgK | GTTTGCCAGGGCTGGAACCAGTGGAACC |
| 5’ IgK | GAGCAGCTAGCATGGAGACAGACACACTCCTGCTATG |
| 5’ IgK-hCC | CAGGTTCCACTGGTTCCAGCCCTGGCAAAC |
| 5’ NheI natlnt | TACGCTAGCATGGCCGGCCCTCTG |
| 5’ Sfi-hCC | ATAGGCCCAGCCGGCCTCCAGCCCTGGCAAACCTC |
| 3’-HCC | TGAATATGCGGCCGCGGCATCCTGACAGGTGG |
